# Predicted effects of hip distraction on knee ligament mechanics following total knee arthroplasty: A finite element analysis

**DOI:** 10.1016/j.clinbiomech.2026.106763

**Published:** 2026-01-24

**Authors:** Sophia Soehnlen, Yogesh Kumaran, Sara Sadeqi, Sudharshan Tripathi, Angela C. Collins, Carmen E. Quatman

**Affiliations:** a Department of Orthopaedics, College of Medicine, The Ohio State University Wexner Medical Center, Columbus, OH, USA; b Engineering Center for Orthopaedic Research (E-CORE), Departments of Bioengineering and Orthopaedic Surgery, The University of Toledo, Toledo, OH, USA; c Department of Emergency Medicine, College of Medicine, The Ohio State University Wexner Medical Center, Columbus, OH, USA

## Abstract

**Background:**

Hip distraction is essential for hip arthroscopy and other orthopaedic surgeries, but high traction forces often lead to complications like nerve injury and soft tissue impairment. Patients with total knee arthroplasties (TKAs) may face increased risk due to altered joint mechanics; however, the impact of specific TKA configurations on knee behavior during distraction is poorly understood.

**Methods:**

A validated finite element model of the pelvis and lower extremity was developed from patient CT and MRI data. Three configurations were modeled: bi-cruciate retaining (BCR), posterior-cruciate retaining (PCR; lacking ACL), and posterior-stabilized (PS; lacking both cruciates). Hip distraction was simulated with axial traction forces (100–500 N) applied to the distal tibia/fibula with the pelvis fixed proximally. Knee and hip joint displacement and ligament strains were evaluated.

**Findings:**

The BCR model demonstrated the highest knee stiffness. ACL removal (PCR) reduced stiffness more compared to PCL removal. While the amount of hip distraction was consistent across models, PCR and PS knees experienced excessive knee distraction (>12 mm) and ligament strains exceeding 20% before achieving 10 mm of hip distraction. Conversely, the BCR model remained under 10 mm of knee distraction and 15% strain at equivalent hip distraction levels.

**Interpretation:**

Finite element analysis revealed that PCR and PS configurations exhibit knee instability, exceeding soft tissue injury thresholds (>20% strain) within standard clinical traction ranges (250–350 N). The ACL contributed greater to knee complex stiffness in axial traction compared to the PCL. Standard traction forces may pose an iatrogenic risk to PCR and PS patients, suggesting a need for TKA-specific distraction protocols to prevent soft tissue failure.

## 1. Introduction

Hip distraction is a versatile technique in minimally invasive hip surgery, enabling treatment of both intra- and extra-articular pathologies such as joint preservation, dysplasia correction, avascular necrosis management, fracture reduction and fixation, and pelvic external stabilization (Casp and Gwathmey, 2018; Sing et al., 2015). Recent advancements in hip arthroscopy, driven by novel instrumentation and refined surgical techniques, have increased its frequency, particularly among older adults, who are both more prone to falls and often have undergone or may require total knee arthroplasty (TKA). These innovations have significantly reduced surgical complications and expedited recovery compared with traditional open methods (Arriaza, 2023; Chung et al., 2020; Marin-Pena et al., 2017). Achieving adequate visualization of the hip joint during surgery often requires mild distraction, accomplished through controlled joint dislocation by applying axial traction. Intraoperatively, the patient’s foot and ankle are securely positioned within a specialized traction boot attached to a sliding mechanism on the surgical table. A perineal post may be used to stabilize the patient’s pelvis, minimizing unintended patient movement when traction is initiated. Upon activation of the device, traction forces pull the lower extremity distally, creating an axial load that gently separates (distracts) the hip joint surfaces. This setup provides the surgeon clear and unobstructed access to the joint space which facilitates improved surgical precision (Byrd, 1994; Mei-Dan et al., 2018).

The reported complication rates for hip arthroscopies vary from 1.4% to 7%, encompassing issues such as transient neurapraxia and iatrogenic soft tissue injury largely attributed to the substantial forces required for hip joint distraction (Casp and Gwathmey, 2018; Jamil et al., 2018; Kowalczuk et al., 2013; Papavasiliou and Bardakos, 2012; Simpson et al., 2010). A systematic review conducted by Arriaza et al. (Arriaza, 2023) found that 13% of complications in 623 assessed hips were directly related to distraction. Factors such as body weight and capsule integrity play crucial roles in influencing the necessary traction force for optimal joint spacing (Arvidsson, 1990; Dienst et al., 2002; Roling et al., 2020). Additionally, pre-existing conditions and prior surgeries of the knee and hip joints can compromise their biomechanical stability, increasing the risk of iatrogenic damage during hip distraction (Truntzer et al., 2017). For instance, Yilmaz et al. (Yilmaz et al., 2015) documented a case where traction for an intertrochanteric hip fracture led to knee dislocation in a patient with pre-existing degenerative knee arthritis and torn cruciate ligaments. Frandsen et al. (Frandsen et al., 2017) also found that nearly half of patients who underwent hip distraction experienced knee-related issues, with 27% reporting knee laxity. This issue is of particular concern in patients who have undergone TKA, where the removal of cruciate ligaments compromises knee stability (Cromie et al., 2008; Sabatini et al., 2021; Verra et al., 2013). Despite these clinical observations, research into the biomechanical impact of distraction forces on the knee joint during surgery remains scarce (Frandsen et al., 2017; Papavasiliou and Bardakos, 2012). However, only one small clinical series (*n* = 9; five TKAs) has examined distraction in lower-extremity arthroplasty patients, limiting generalizability (Beutel et al., 2015). No biomechanical investigations of TKA knees under hip distraction exist, emphasizing the need for a parametrized finite element analysis (FEA).

The present study seeks to bridge this knowledge gap by investigating the biomechanical behavior of the knee joint under the influence of simulated traction forces, especially in patients with a history of TKA and ligament removal. Through a comprehensive parametrized FEA, we aim to understand the kinematics of the knee joint and the strain on ligaments during hip distraction after various TKA techniques.

## 2. Methods

### 2.1. Model development

Geometries from a previously validated finite element (FE) model of the lower extremity was utilized in this study to further validate its use under intraoperative loading conditions (Ingels, 2016). The bony structures and main ligament geometries of a 45-year-old female were obtained from computed tomography and magnetic resonance imaging, respectively. Mimics v15 (Materialise, Leuven, Belgium) was used for segmenting the medical images. The 3D reconstructed tissues were imported into Geomagic Studio (3DSystems, Morrisville, North Carolina, USA) for further smoothing and partitioning (Supplemental Fig. 1). Three-dimensional geometries for TKA instrumentation (Supplemental Fig. 2) were created using Solidworks computer aided design (Dassault Systèmes, Vélizy-Villacoublay, France). Blender (Blender foundation) was utilized to perform Boolean operations to remove the bony features of the 3D geometry where the implant was placed. Following a previous study (Ingels, 2016), the TKA was implanted in the computational model under guidance of an orthopaedic surgeon (Supplemental Fig. 1). Meshlab (Cignoni et al., 2008) was used to apply surface meshes to each component which were converted into C3D4 tetrahedral elements in Abaqus 2019 (Simulia, Providence, Rhode Island, USA). A mesh convergence study was performed on the components of the model to ensure proper mesh density and quality. Mesh size was consecutively iterated by 0.25 mm, increasing the total number of elements. The parts were subjected to the same loading and boundary conditions and the maximum von Mises stress was recorded. The mesh density was chosen by selecting the element size for each category that fell within 5% of the most densely meshed trial (Supplemental Table 1–3). The meshed components were then assembled in Abaqus 2019.

Hyperelastic and linear elastic materials were used to define the various structures of the model (Supplemental Table 4.1 and 4.2) (Ingels, 2016; Kiapour et al., 2014). In the knee joint, the anterior cruciate ligament (ACL), posterior cruciate ligament (PCL), medial collateral ligament (MCL), and lateral collateral ligament (LCL) were modeled with hyperelastic material models (Sadeqi et al., 2021). Peters et al. (Peters et al., 2022) reported experimental stress-strain data of the knee ligaments for a range of ages in males and females. To age-match the material properties closely to the current model, the experimental data from a healthy 43-year-old female was utilized. A hyperelastic, neo-Hookean material model was applied to the ACL, PCL, MCL, and LCL (Mootanah et al., 2014). The capsule ligaments and muscles of the knee complex were modeled using truss elements with linear elastic material properties similar to a previous studies conducted by Erbulut et al. (Erbulut et al., 2021).

### 2.2. Model validation

Validation practices were performed based on the guidance provided by the American Society of Mechanical Engineering (ASME) VVUQ 40–18 (ASME, 2018). To validate the hip joint under traction forces, hip joint dislocation was compared against cadaveric hip load-displacement data (Stewart et al., 2002). The pelvis was fixed, and an axial load was applied distally on the femur, at the intercondylar fossa (ASME, 2018). Frictionless surface to surface interactions with exponential pressure-overclosure behavior were used to model the hip joint based on a previous FE study (Joukar et al., 2019). The hyperelastic, Yeoh material model was used to model the hip capsule (Supplemental Fig. 3) as suggested by Stewart at el (Stewart et al., 2004; Yeoh, 1993). Yeoh material coefficients (Supplemental Table 5) were determined via an iterative calibration process. Coefficients were adjusted parametrically to minimize the deviation between the experimental load-displacement bounds reported in the literature (Stewart et al., 2002; Stewart et al., 2004; Yeoh, 1993), ensuring the model accurately captured the characteristic non-linear stiffness of the capsule (Supplemental Fig. 4).

The stiffness of the knee ligament complex was validated against clinical data reported for ligament balancing using the DePuy Synthes Knee Balancer (DePuy Synthes, Warsaw, IN, USA) in PCR TKAs. For the validation study, the distal portion of tibia and fibula was encastered to restrict translations and rotations. Using SolidWorks, metal paddles resembling the Knee Balancer device were created and placed on the femoral condyles as directed by device protocol (Vollner et al., 2019a). An axial force was applied on each paddle similar to the device mechanism described by Völlner et al. (Vollner et al., 2019a). Force displacement curves were constructed and compared against previously collected clinical data that used the same device (Vollner et al., 2017; Vollner et al., 2019a).

### 2.3. Simulations

**Fig. 1.**
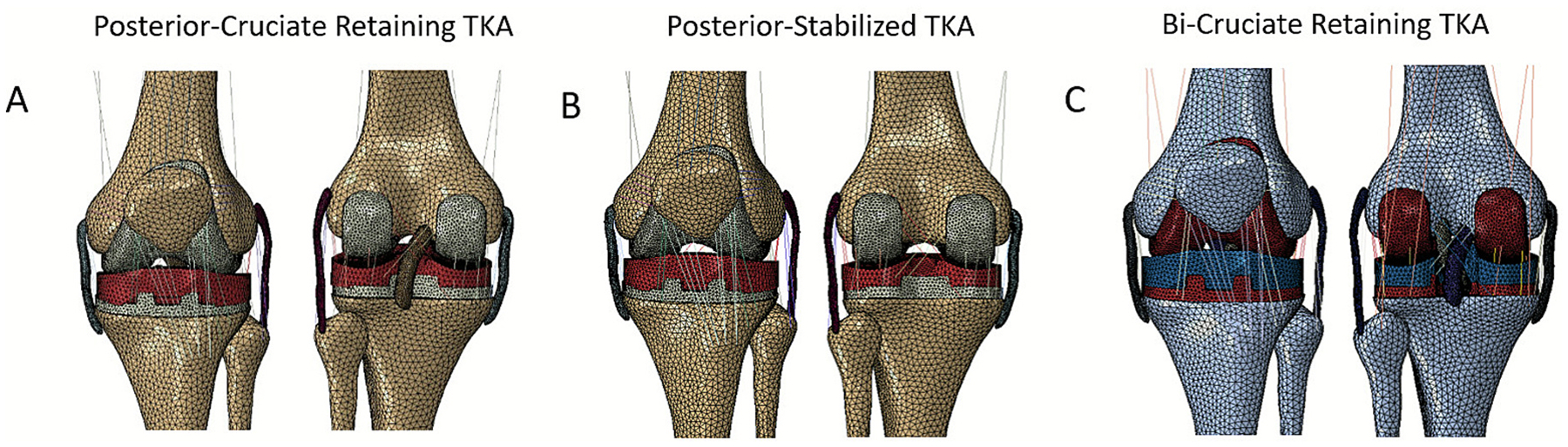
Finite element knee models illustrating ligament status across total knee arthroplasty (TKA) configurations. A) Posterior-cruciate retaining (PCR) TKA with simulated anterior cruciate ligament (ACL) release. B) Posterior-stabilized (PS) TKA with simulated ACL and posterior cruciate ligament (PCL) releases. C) Bicruciate retaining (BCR) TKA with intact ACL, PCL, medial collateral ligament (MCL), and lateral collateral ligament (LCL). Absent ligaments reflect simulated surgical release. Anterior and posterior views are shown for each configuration.

Three different models were assembled according to different TKA techniques performed: Bi-Cruciate Retaining (BCR) model, Posterior-Cruciate Retaining (PCR) model, and the Posterior Stabilized (PS) model (Fig. 1A–C). The BCR model retains all native ligaments of the knee joint (ACL, PCL, MCL, and LCL), whereas the PCR model was subject to ACL removal and the PS model was subject to ACL and PCL removal (D’Anchise et al., 2013). The TKA instrumentation was assumed to be perfectly bonded and modeled with a rigid “tie” interaction at the bone interface to restrict micromotion between implant and bone. Frictionless, hard contact surface to surface interactions were applied at the patellofemoral and tibiofemoral joints. The hip was joint was positioned at 10^0^ flexion and 0^0^ abduction under the guidance of an orthopaedic surgeon to fall within the range of previously reported positioning of the lower extremity (Supplemental Fig. 1) (Byrd, 1994; Mannava et al., 2015). The pelvis was encastered to prevent its motion under the traction forces as motion of the patient’s trunk is restrained, intraoperatively (Byrd, 1994). To simulate the loading condition of hip distraction, an axial force was coupled to the distal fibula and tibia (Mannava et al., 2015; Metz et al., 2022). Clinical studies have indicated a wide range of required traction forces (100 N–600 N) and a recommended minimum of 10 mm of hip joint distraction (Ellenrieder et al., 2017; Eriksson et al., 1986; Griffiths and Khanduja, 2012; Grontvedt and Engebretsen, 1995; O’Neill et al., 2021; Roling et al., 2020); thus, the present model was subjected to traction forces ranging between 100 N to 500 N to analyze joint behavior. Simulations were performed using the Abaqus Standard implicit solver to calculate static equilibrium at each force increment.

### 2.4. Data analysis

Joint spacing and ligament strain were analyzed to assess the effects of traction forces. Relative strain (i.e. strain relative to pre-traction length) was calculated following methodology previously reported (Sadeqi et al., 2023). To measure the compartmental knee joint spacing, two nodes on the FE mesh vertical to each other were selected in the center region of the tray component and femoral component of the TKA system for the medial and lateral compartments. The difference before and after traction was measured to calculate the knee joint spacing. For hip joint distraction, a point selected on the femoral head near the centroid was used to calculate the displacement of the femur respective to the hip joint as suggested by a previous study (Kapron et al., 2018). Statistical analysis and graphs were produced using MATLAB (Math-Works, Massachusetts, USA).

## 3. Results

### 3.1. Validation

**Fig. 2.**
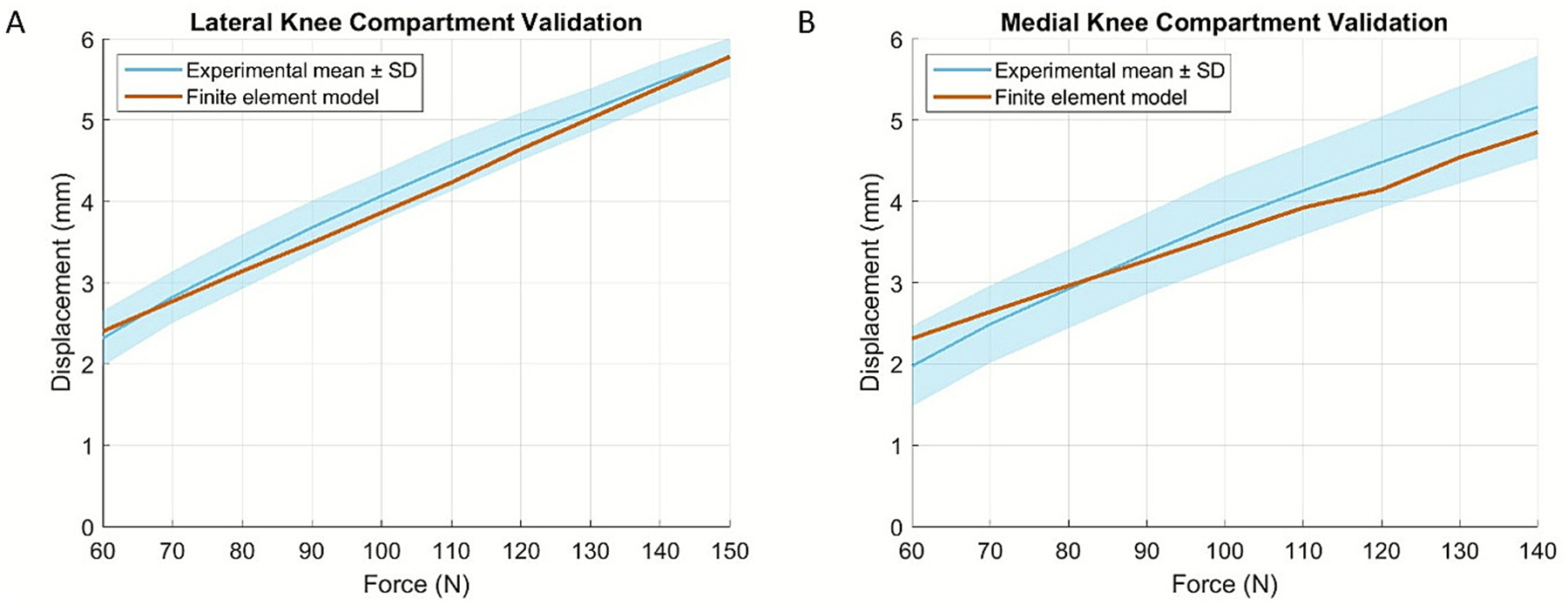
Knee ligament balancing for the A) lateral and B) medial compartment for PCR TKAs. The blue lines and shaded regions represent cadaveric and in-vivo reported load-displacement values (Vollner et al., 2017; Vollner et al., 2019a). The orange line represent the load-displacement data for the PCR FE model under knee ligament balancing.

The FEA simulation of hip dislocation fell within an agreeable range of previously measured cadaveric data (Supplemental Fig. 4). Moreover, the PCR model exhibited joint stiffness values of 25.7 N/mm (lateral compartment) and 27.8 N/mm (medial compartment), showing excellent agreement with clinical benchmarks. As detailed in Supplemental Table 6, these values fall within the reported clinical range in the literature (lateral compartment: 19.9–31.6 N/mm; medial compartment: 25.7–28.4 N/mm).The trend data for ligament balancing during a PCR TKA in Fig. 2 fell within range of in-vivo data (Stewart et al., 2002; Stewart et al., 2004).

### 3.2. Hip joint distraction

**Fig. 3.**
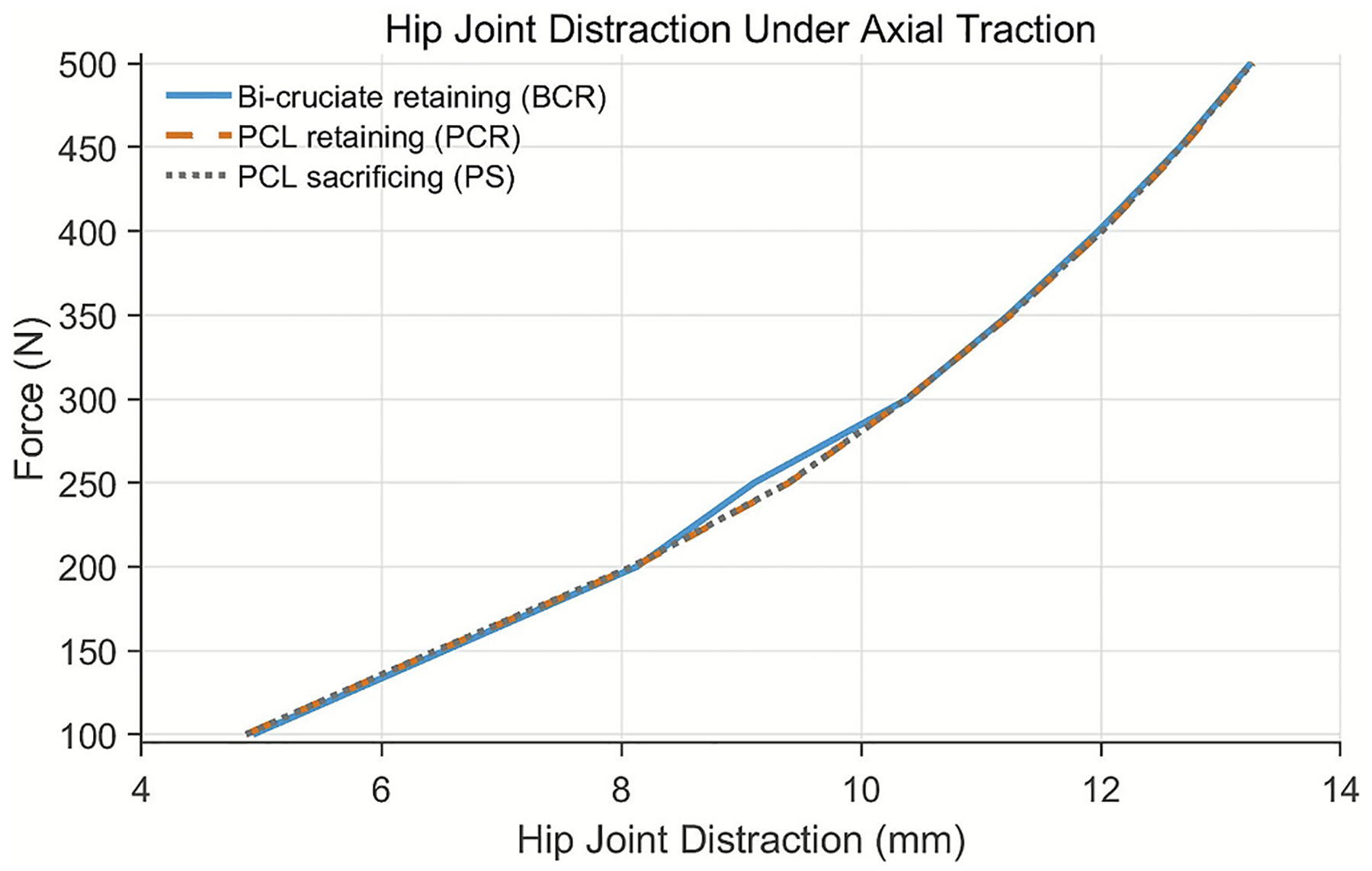
Hip joint distraction under axial traction forces for varying TKA implant scenarios (BCR, PCR, and PS). The overlapping curves indicate that the different ligament sacrifice states associated with these TKA designs minimally impact the amount of distraction occurring at the hip joint.

Based on the clinical suggestion of 10 mm of hip joint spacing, adequate joint visualization for the present model was achieved approximately between 250 N to 350 N (Kapron et al., 2018). Fig. 3 displays the hip joint distraction for each of the implant scenarios. Across the entire loading spectrum, the maximum percent difference in femoral head displacement between the three implant models was 2.7% (observed at 250 N). At the clinical visualization threshold of 300 N, this variability decreased to less than 0.5% (Fig. 3).

### 3.3. Knee complex

**Fig. 4.**
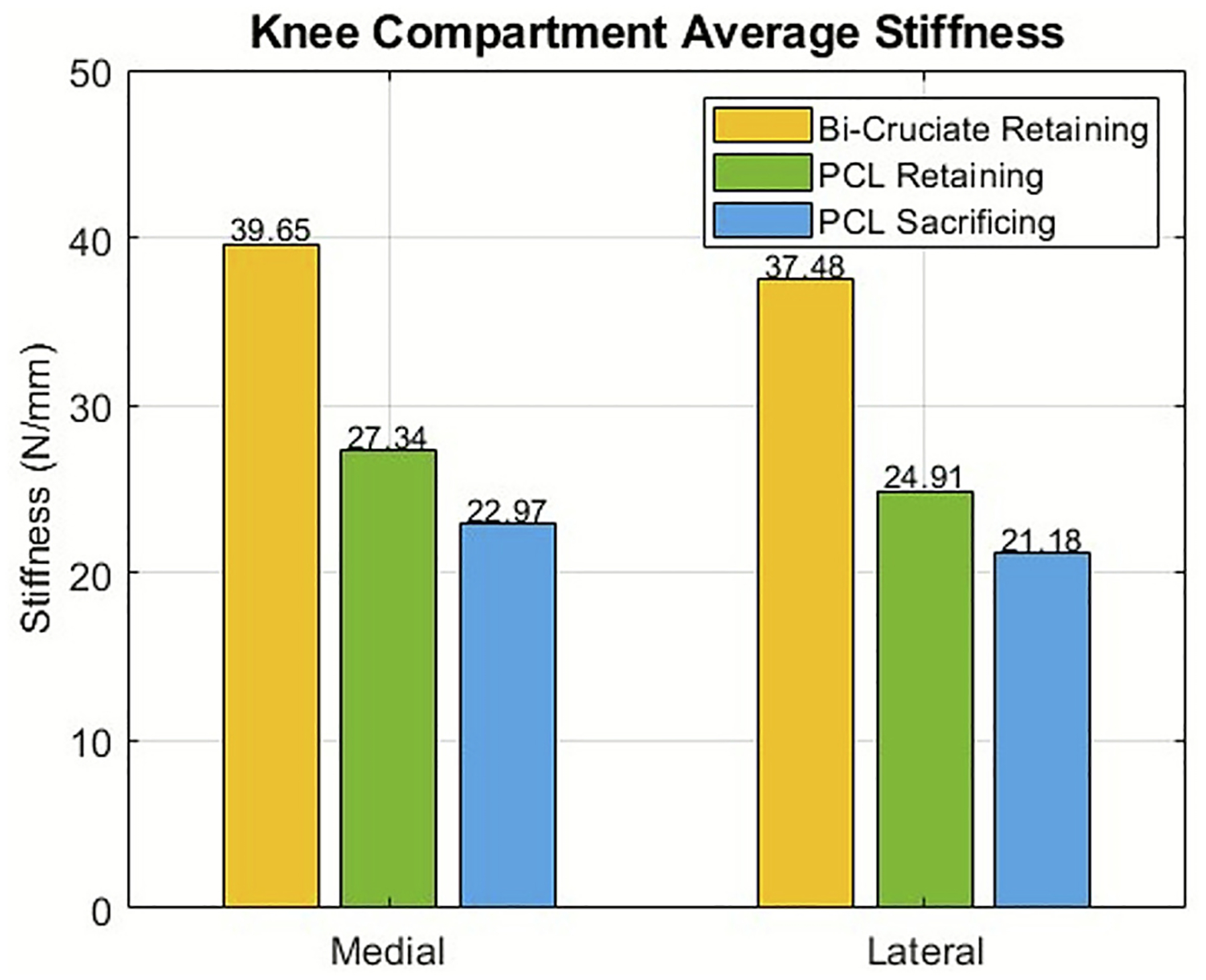
Average stiffness for the knee complex in Bi-cruciate retaining (BCR), PCL retaining (PCR), and PCL sacrificing (PS) during the range of distraction forces.

The lateral and medial compartment stiffness of the knee joint was analyzed under hip distraction for the three different TKA scenarios. The BCR model displayed the greatest average knee complex stiffness of 39.65 N/mm for the medial compartment and 37.48 N/mm for the lateral compartment compared to the PCR and PS implants (Fig. 4). With release of the ACL for the PCR implant, there was a 31% and 34% decrease in the medial and lateral compartment stiffness, respectively, compared to the intact BCR model. Release of the PCL and ACL for the PS implant resulted in a stiffness decrease of 42% medially and 43% laterally compared to the BCR, and a 16% and 15% decrease in medial and lateral compartment stiffness compared to the PCR case.

**Fig. 5.**
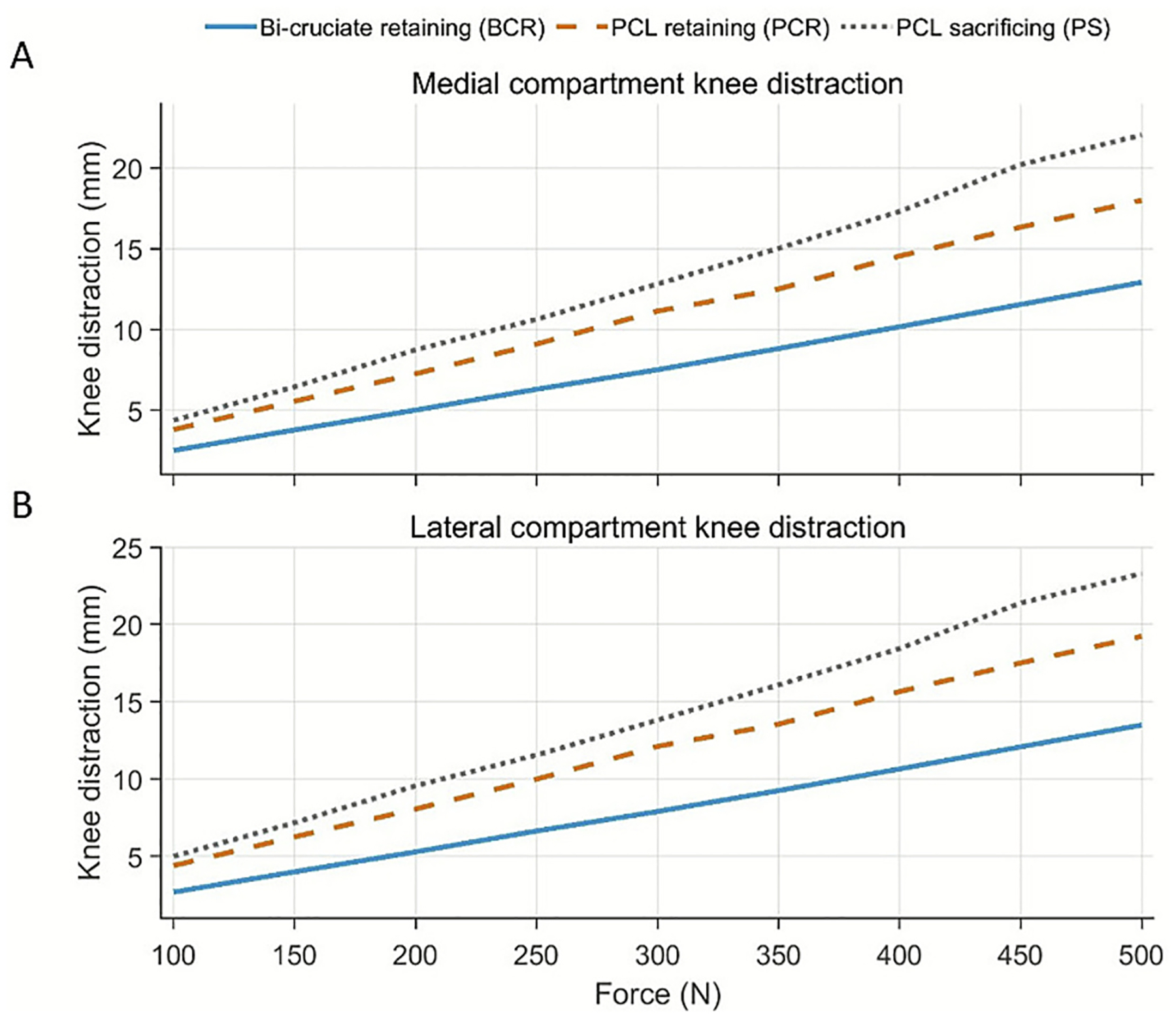
Knee joint distraction under the axially applied traction forces for the A) medial and B) lateral compartment.

The knee joint displacement during distraction was recorded. The PS implant resulted in the greatest knee joint spacing at each force, whereas the BCR model had the least amount of joint spacing for both compartments (Fig. 5). On average, the PCR model’s medial compartment resulted in a 45% greater joint spacing compared to the BCR model, and the PS model resulted in a 72% increase in medial compartment spacing.

### 3.4. Knee ligament strain

**Fig. 6.**
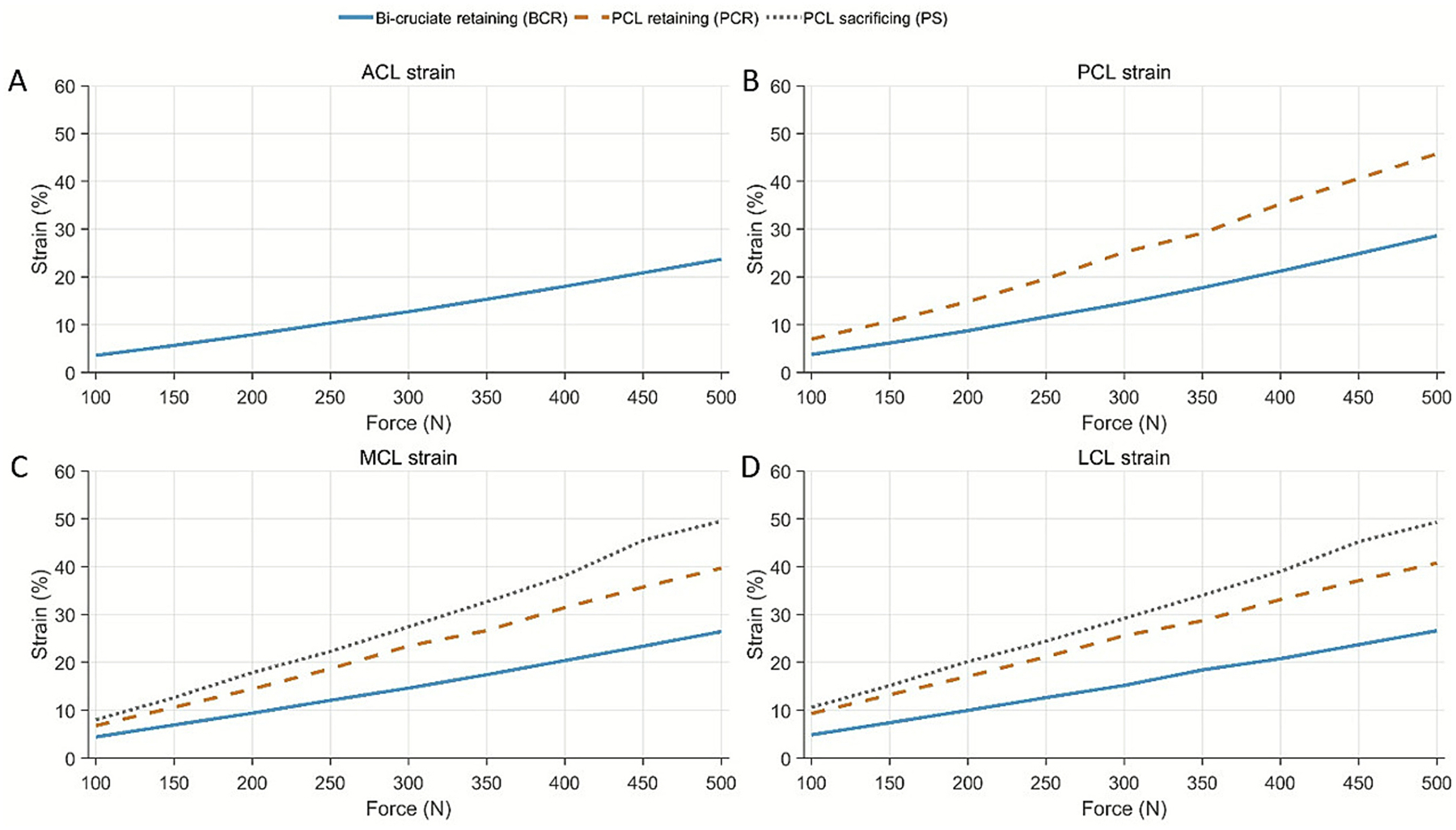
Measured strain in the A) ACL, B) PCL, C) MCL, and D) LCL under the traction forces.

The relative strain of the four main ligaments was measured under traction forces ranging from 100 N to 500 N. The MCL displayed the greatest sensitivity to TKA technique, exhibiting the highest peak strain of 49.49% in the PS model (at 500 N) and the lowest peak strain of 26.45% in the BCR model. In comparison, the LCL followed a similar trend ranging from a peak of 26.63% (BCR) to 49.30% (PS) (Fig. 6).

The ACL demonstrated the greatest stability among the ligaments in the BCR model, maintaining the lowest strain at every force increment, starting at 3.58% (100N) and reaching only 23.69% at maximum load. In contrast, the PCL endured higher loads when the ACL was sacrificed; PCL strain increased from a peak of 28.61% in the BCR model to 45.74% in the PCR model (Fig. 6).

Comparing the surgical models, the removal of the ACL (transitioning from BCR to PCR) resulted in an increase in collateral ligament strain. At maximum load, MCL strain rose from 26.45% to 39.70%, while LCL strain increased from 26.63% to 40.76%. The further removal of the PCL (transitioning from PCR to PS) caused an additional, though smaller, spike in strain, with the MCL and LCL reaching 49.49% and 49.30%, respectively. However, the strain for the MCL and LCL for the PS model compared to the BCR model increased by 87% and 95%, respectively (Fig. 6).

## 4. Discussion

In this study, the posterior cruciate-retaining (PCR) and posterior-stabilized (PS) configurations exhibited greater knee joint distraction and higher ligament strains before achieving adequate hip separation, whereas the bicruciate-retaining (BCR) model consistently showed the lowest levels of both distraction and strain. While it is intuitive that sacrificing ligaments increases laxity, our analysis identifies that the mechanical divergence between TKA techniques is not linear; rather, it manifests specifically at the distraction forces necessary for hip visualization (250–350 N), where PCR and PS models exceeded injury thresholds (>20% strain). These results may help explain clinical reports of post-operative pain and instability in the knee joint (Arriaza, 2023). Furthermore, we found that the PCL contributes minimally to resisting axial distraction. We observed only a 15–16% difference in stiffness between PCR and PS models, with minimal corresponding strain increases in the MCL (18%) and LCL (15%) upon PCL release. Collectively, these findings reveal how varying TKA techniques alter knee mechanics within a validated framework (Ellenrieder et al., 2017; Stewart et al., 2002; Stewart et al., 2004; Vollner et al., 2017; Vollner et al., 2019a; Vollner et al., 2019b; Yin et al., 2023), delineating the specific force ranges where iatrogenic injury may occur.

The stiffness of the hip joint (Supplemental Fig. 4) was shown to be greater than that of the knee complex subjected to PCR and PS TKAs (Fig. 4). Previous literature supports these findings with the hip capsule stiffness ranging between 12.7 N/mm to 60.2 N/mm based on ligament section (Stewart et al., 2002). However, there is limited knowledge of the intact stiffness of the knee complex under traction forces (Vollner et al., 2019b). Our results from the BCR model with intact knee ligaments report a stiffness of 39.65 N/mm and 37.78 N/mm for the medial and lateral compartments, respectively. Based on the previous literature findings for hip joint stiffness and our model predictions, the lower stiffness of the knee joint may predispose it to negative effects from hip distraction forces. Moreover, we observed a large reduction in knee complex stiffness following ligament releases for PCR and PS TKAs (Fig. 4). Ligament balancing, aimed at correcting varus and valgus deformities in TKAs patients, often involves sequential ligament releases in PCR TKAs. Yet, literature advises against complete MCL releases due to its critical role in passive knee stabilization (Vollner et al., 2019b) with stiffness potentially decreasing by up to 40% post-release (Vollner et al., 2019b). Based on the trends outlined in the present study, patients undergoing additional ligament releases may be at an increased risk for traction-related injury during hip distraction.

The contribution of the PCL to knee stability under traction force remains a topic of debate, with no consensus on its biomechanical role when the knee is fully extended (Matsueda et al., 1999; Vollner et al., 2019b). Fig. 5 suggests a relationship between the PCL’s contribution to stability and the applied traction force. Our results indicate that the PCL’s role in passive stabilization is comparatively minor, with only a 16% and 15% difference in stiffness in medial and lateral compartments, respectively, observed between the PCR and PS models. Additionally, the MCL and LCL had minimal increase in strain (18% and 15%, respectively) when the PCL was released. This emphasizes the predominant stabilizing functions of the ACL.

Our results indicated that knee complex stiffness varied based on the intraoperative technique employed, showing an average increase in joint spacing of 45% for PCR and 72% for PS models relative to the BCR model (Fig. 5). While injury can occur within a wide range of strain values depending on patient specific factors, there is limited data quantifying ligament strains in intact knee kinematics (Butler et al., 1986; Quapp and Weiss, 1998). In the present study, within the traction force ranges of 250 N to 350 N needed for adequate joint visualization, the strain for the MCL and LCL was greater than 20% for the PCR and PS simulations which may suggest potential for ligament damage (Butler et al., 1986). The BCR model resulted in strain levels that remained below 20%. Moreover, when knee joint spacing exceeds 10 mm of distraction, there may be risk for ligament failures and iatrogenic dislocation due to high strain values noted in Fig. 6. The BCR technique may reduce the chance of traction related complications because retaining the native ligaments offers biomechanical advantage. However, the BCR technique has been debated due to poor surgical outcomes with unclear indications for use, making it a less widely used technique and implant configuration compared to the PCR technique (Trecci, 2016). In one study of a small cohort, only 16% of patients received a BCR with the main indication of use being an intact ACL (Christensen et al., 2017). The strain values noted in the BCR model were within reasonable range compared to strain values reported during native knee motion, suggesting a minimal risk of injury (Hosseini Nasab et al., 2016; Taylor et al., 2013). However, when surpassing the recommended 10 mm of distraction at 300 N of traction, the strain levels in the ACL were 12.7%, suggesting minor injury may occur (Butler et al., 1986).

The FE findings, which show large changes in knee complex stiffness and joint spacing, align with clinical observations reported in the literature, where approximately 50% of patients experience sensations of instability and pain in the knee following procedures involving hip distraction (Frandsen et al., 2017). Clinically, our results highlight the potential heightened risk of excessive ligament strain in TKA patients subjected to standard boot-and-post traction. In revision prostheses, particularly hinged knee systems that lack native ligament constraints, this traction modality may prove hazardous, suggesting that through-bone methods (e.g., sterile femoral traction pins) used in fracture fixation could offer a safer alternative in non-arthroscopic settings. In response, various surgical procedures have emerged to reduce traction force and time (Gupta et al., 2014; O’Neill et al., 2023). Lateral or supine patient positioning are used during hip arthroscopy, but the superiority of one over the other is debated (de Sa et al., 2016). Despite the approach used, abduction and flexion of the hip are used to relax the hip capsule to allow for easier distraction (Dienst et al., 2002; Glick et al., 1987; Lall et al., 2019). Additionally, various surgical interventions have been proposed to alter the resistive forces of the hip capsule. Distension combined with traction increases joint distraction upwards of 2.25 times compared to traction alone (Byrd and Chern, 1997; Dienst et al., 2002). Furthermore, joint venting prior to the application of traction significantly reduces the amount of traction needed, however, the required force still fell within the range of 100 N to 450 N (Mortensen et al., 2023; O’Neill et al., 2021). Based on the trends in the present study, the force required remains in a harmful range for patients with PCR and PS TKAs. Moreover, capsulotomies have been a debated procedure to reduce traction forces (Khair et al., 2017; Perry et al., 2021). The size of the capsulotomy is known to reduce the traction force; however, capsule repair is essential to return the capsule to its native functionality (Hartwell et al., 2023; Weber et al., 2018). The combination of joint venting and capsulotomies further reduces the traction force safely, but the lower extremity joints remain exposed to excessive traction (O’Neill et al., 2021; Roling et al., 2020). Further research and innovative devices are needed to study and combat hip distraction forces for vulnerable patient populations (Soehnlen et al., 2025).

### 4.1. Limitations

There are limitations in this study that require discussion. Model development was constrained by the availability of validated experimental data for parameterization and verification. Simulations were limited to TKA states to maintain consistency with validation evidence, as relevant cadaveric distraction data were only available for TKA specimens. Within this framework, the BCR model, which preserves all major native stabilizing ligaments, was included to be interpreted as a ligament intact reference. Future studies should incorporate native knee distraction data to quantify differences between intact and arthroplasty states and to refine clinical distraction and strain thresholds.

Our findings highlight the role of the ACL in knee stabilization during distraction forces, it’s important to note that the material properties used in our analysis were derived from a healthy, non-arthritic individual. Given the known susceptibility of intraarticular ligaments to degradation from osteoarthritis (OA) and aging (Peters et al., 2022; Schulze-Tanzil, 2019) our study’s application of semi-patient-specific material properties may not fully encapsulate the biomechanical realities of healthy patients or those with advanced OA. Despite this limitation, our results indicate that individuals with a history of TKA, potentially exhibiting compromised ACL and PCL properties due to OA, could be at an elevated risk of injury during surgery.

In addition, the experimental knee data used for model validation were obtained intraoperatively with the joint capsule opened. We acknowledge this represents a mechanically more compliant condition than an intact or post-arthroplasty knee with a closed capsule. However, we were constrained by the limited availability of quantitative in vivo data, and these intraoperative measurements remain the only existing studies quantifying distraction in this specific state. Therefore, utilizing this data allowed us to validate the model against the best available clinical standard. As a result, the knee distraction and ligament strain observed in the simulated TKA configurations may represent lower bound estimates of in vivo stiffness.

Our approach to modeling muscle forces as uniaxial truss-connector elements with linear, elastic material properties, while simplified (Kiapour et al., 2014), closely mirrors the clinical scenario during hip distraction procedures where patients are under general anesthesia. In this state, muscles function as passive stabilizers, akin to the passive mechanical behavior assumed in our simulations. This aspect of our model is relevant given that, under anesthesia, the dynamic and active properties of muscle forces are substantially reduced. A previous study highlighted increased hip traction forces required in awake patients (Eriksson et al., 1986), a situation which diverges from standard practice where anesthesia is employed, reinforcing the clinical applicability of our modeling assumptions. Rather than detracting from the study’s validity, this simplification may enhance the relevance of our findings to the typical clinical environment.

Lastly, the PS model was simplified by using the PCR model with a PCL release. While simplified in the present study, a PS implant commonly has a uniquely designed cam-post that limits anterior-posterior translation. However, this would not alter the mechanism under traction forces (Murakami et al., 2018).

## 5. Conclusions

While traction forces are a significant concern for patient outcomes in hip distraction procedures, the biomechanical impact on the lower extremity post-TKA has not been fully explored. Our models show that PCR and PS configurations exhibit knee stability, with joint spacing increasing by 45% and 72% compared to BCR models. Critically, within the clinical traction range of 250–350 N, both PCR and PS models exceeded the soft tissue injury threshold (>20% strain), while the BCR model only reached 12.7% ACL strain at loads exceeding 300 N. Furthermore, retaining the PCL may offer minimal protective benefit against axial distraction, contributing only a 15–16% increase in stiffness over the PS model. Consequently, standard traction forces may induce iatrogenic soft tissue failure in PCR and PS patients. These quantitative insights point towards the necessity of further research into hip distraction to refine safety protocols and enhance patient care.

## Supplementary Material

1

## Appendix A. Supplementary data

Supplementary data to this article can be found online at https://doi.org/10.1016/j.clinbiomech.2026.106763.
