# Supplementary material for: Predicted effects of hip distraction on knee ligament mechanics following total knee arthroplasty: A finite element analysis
Source: Clin Biomech (Bristol). Author manuscript; Available in PMC 2026 Aug 12. (PMC13465713; doi:10.1016/j.clinbiomech.2026.106763)
Supplement: Supplementary file 1 [file NIHMS2190201-supplement-1.docx]

Supplemental Data

**Supplemental Figure 1.** Lower extremity FE model.

B

A

**Supplemental Figure 2**. Meshed parts of the A.) Posterior Cruciate retaining total knee replacement system and the B.) Bi-cruciate retaining total knee replacement system.

**Supplemental Figure 3**. Hip capsule geometry and anatomical attachment sites based on Stewart et al [32].

**Supplemental Figure 4.** Validation of hip joint load-displacement behavior under axial distraction. Gray lines show experimental load-displacement curves from a single cadaveric specimen across three distraction cycles, measuring hip joint separation [32]. The red line represents the FE simulation using Yeoh hyperelastic coefficients assigned to the hip capsule optimized to the experimental response

## Mesh Convergence

A component from each of the bones, implant, and ligaments geometries, were utilized in the mesh convergence study to appropriately assign mesh density. The maximum Von Mises stress was recorded for varying mesh sizes. For the bone components (Supplemental Table 1), a mesh size of 3 was selected. A mesh size of 1 was selected for the 3D soft tissue components (ligaments and hip capsule) (Supplemental Table 2). Moreover, a mesh size of 2 was selected for each of the TKA implant components (Supplemental Table 3).

| # of Elements | Mesh Size (mm) | Max Von Mises (MPa) |
| --- | --- | --- |
| 297541 | 2 | 1.851 |
| 225953 | 2.25 | 1.827 |
| 173626 | 2.5 | 1.824 |
| 125185 | 2.75 | 1.8 |
| 100724 | 3 | 1.782 |

**Supplemental Table 1**. Mesh convergence study of the femur under tensile load of 50N.

**Supplemental Table 2**. Mesh convergence study of the LCL ligament under tensile load of 50N.

| # of Elements | Mesh Size (mm) | Max Von Mises (MPa) |
| --- | --- | --- |
| 17285 | 0.75 | 3.703 |
| 7495 | 1 | 3.695 |
| 4034 | 1.25 | 3.683 |

**Supplemental Table 3**. Mesh convergence study of the tibial component under tensile load of 50N.

| # of Elements | Mesh Size (mm) | Max Von Mises (MPa) |
| --- | --- | --- |
| 126727 | 1 | 2.888 |
| 77747 | 1.25 | 3.013 |
| 46128 | 1.5 | 3.072 |
| 29369 | 1.75 | 3.099 |
| 20793 | 2 | 3.034 |

## Material Properties

**Supplemental Table 4.1**. Implant material properties [22]

| **Part** | **Material** | **Young’s Modulus (MPa)** | **Poisson’s Ratio** |
| --- | --- | --- | --- |
| Femoral Component | Titanium | 120,000 | 0.3 |
| Tibial Tray | Polyethylene | 690 | 0.46 |
| Tibial Component | Titanium | 120,000 | 0.3 |
| Patellar Dome | Polyethylene | 690 | 0.46 |

**Supplemental Table 4.2**. Hard tissue material properties [22]

| **Material** | **Young’s Modulus (MPa)** | **Poisson’s Ratio** |
| --- | --- | --- |
| Cortical Bone | 15,000 | 0.3 |
| Cancellous Bone | 400 | 0.3 |

**Supplemental Table 5**. Hyperelastic Yeoh material model coefficients for the hip capsule [33].

| **Yeoh** **Coefficients** | C10 | C20 | C30 | D1 | D2 | D3 |
| --- | --- | --- | --- | --- | --- | --- |
|  | 0.65 | 0.75 | 6.85 | 0.000887 | 0.000487 | 0.000487 |

**Supplemental Table** **6**. Knee compartment stiffness for PCR TKAs during knee ligament balancing. The clinical measurements were taken with the DePuy Knee Balancer System (DePuy Synthes, Johnson and Johnson, Raynham, MA). The compartment stiffness in the model was averaged across a range of simulated forces used in knee ligament balancing.

| **Knee Compartment Stiffness** | | |
| --- | --- | --- |
|  | Lateral compartment (N/mm) | Medial compartment (N/mm) |
| **Cadaveric [35]** | 31.6 | 26.6 |
|  |  |  |
| ***In vivo* [34, 35]** | 25.3 | 25.7 |
|  | 19.9± 0.9 | 28.4 ± 1.2 |
|  |  |  |
| **FE Model** | 25.7 | 27.8 |

**Supplemental Table 7**. Table of Nomenclature

| **Abbreviation / Symbol** | **Definition** |
| --- | --- |
| **Anatomical Structures** |  |
| ACL | Anterior Cruciate Ligament |
| LCL | Lateral Collateral Ligament |
| MCL | Medial Collateral Ligament |
| PCL | Posterior Cruciate Ligament |
| **Surgical & Clinical Terms** |  |
| BCR | Bi-Cruciate Retaining (TKA configuration) |
| PCR | Posterior-Cruciate Retaining (TKA configuration) |
| PS | Posterior-Stabilized (TKA configuration) |
| TKA | Total Knee Arthroplasty |
| OA | Osteoarthritis |
| **Modeling & Engineering** |  |
| ASME | American Society of Mechanical Engineers |
| C3D4 | 4-node linear tetrahedron element (Abaqus element type) |
| CT | Computed Tomography |
| FE | Finite Element |
| FEA | Finite Element Analysis |
| VVUQ | Verification, Validation, and Uncertainty Quantification |
| **Variables & Units** |  |
| C_10_, C_20_, C_30_ | Yeoh hyperelastic material model coefficients |
| D_1_, D_2_, D_3_ | Yeoh material compressibility coefficients |
| mm | Millimeters (Unit of length/displacement) |
| MPa | Megapascals (Unit of stress/modulus) |
| N | Newtons (Unit of force) |
| N/mm | Newtons per millimeter (Unit of stiffness) |
